# Efficacy of prehospital amiodarone on survival in adult out-of-hospital cardiac arrest: a retrospective observational study

**DOI:** 10.62838/jccm-2026-0024

**Published:** 2026-07-27

**Authors:** Yuki Kishihara, Shunsuke Amagasa, Yosuke Homma, Takashi Tagami, Hideto Yasuda, Masahiro Kashiura, Yutaro Shinzato, Takashi Moriya, SOS-KANTO 2017 Study Group

**Affiliations:** Department of Emergency and Critical Care Medicine, Jichi Medical University Saitama Medical Center, Saitama, Japan; Department of Emergency and Transport Medicine, National Center for Child Health and Development, Tokyo, Japan; Department of Emergency and Critical Care Medicine, Chiba Kaihin Municipal Hospital, Chiba, Japan; Department of Emergency and Disaster Medicine, The Jikei University School of Medicine, Tokyo, Japan; Department of Clinical Research Education and Training Unit, Keio University Hospital Clinical and Translational Research Center, Tokyo, Japan; School of Nursing and Midwifery, Alliance for Vascular Access Teaching and Research, Griffith University,Australia; School of Nursing, Midwifery and Social Work, UQ Centre for Clinical Research, The University of Queensland,Australia; Department of Emergency Medicine, Teikyo University School of Medicine, Tokyo, Japan

**Keywords:** amiodarone, cardiopulmonary resuscitation, emergency medical services, out-of-hospital cardiac arrest

## Abstract

**Aim of the study:**

This study sought to determine whether prehospital administration of amiodarone improves outcomes among adult patients with out-of-hospital cardiac arrest (OHCA) presenting with ventricular fibrillation (VF) or pulseless ventricular tachycardia (VT). The analysis accounted for time-dependent confounding and resuscitation time bias using real-world registry data.

**Material and methods:**

We conducted a multicenter, retrospective cohort study using a nationwide Japanese OHCA database, including adult nontraumatic shockable rhythms. The exposure was prehospital amiodarone administration at a given time, and the comparator was no amiodarone at the same time point. The comparison reflects amiodarone administration at that time versus no amiodarone, not ‘amiodarone versus never-amiodarone’. The primary and secondary endpoints were favorable neurological status and survival at 30 days. Patients were matched 1:3 using time-dependent propensity score matching, followed by generalized estimating equations to address intrahospital clustering. Sensitivity analyses included covariates with standardized mean differences greater than 0.25 after matching. Associations were expressed as risk ratios (RRs) with 95% confidence intervals (CIs).

**Results:**

Among 9,909 eligible patients, 56 (0.6%) remained after matching, including 19 (0.2%) who received amiodarone at the index time point. Median (IQR) age was 65 (52–76) years, and 81.8% were male. The median interval from first medical contact to drug administration was 27 (22–32) minutes. In models adjusted for timing variables and hospital clustering, amiodarone was not significantly related to favorable neurological recovery (RR, 0.45 [95% CI, 0.14–1.47]) or survival (RR, 0.74 [95% CI, 0.31–1.73]). Sensitivity analyses yielded consistent findings, though survival model convergence was limited.

**Conclusions:**

Prehospital administration of amiodarone in adult OHCA patients with shockable rhythms was not associated with improved neurological or survival outcomes. However, these findings should be interpreted with caution, and further studies are warranted to confirm and extend these observations.

## Introduction

Out-of-hospital cardiac arrest (OHCA) remains a leading cause of mortality across the globe [1]. Approximately 20–30% of these events present with ventricular fibrillation (VF) or pulseless ventricular tachycardia (VT), collectively classified as shockable rhythms [2]. It is well established that patients with OHCA exhibiting a shockable rhythm have a more favorable prognosis than those presenting with nonshockable rhythms [3,4,5]. In contrast, persistent or recurrent VF or pulseless VT is associated with poorer clinical outcomes [6,7]. Therefore, rapid defibrillation is fundamental to improving survival among patients with shockable rhythms, and amiodarone is frequently used when VF or pulseless VT proves refractory to shocks [8,9,10].

The potential benefit of administering amiodarone before hospital arrival in shock-refractory OHCA has been examined in two randomized controlled trials (RCTs) [11,12]. In one study, patients with VF or pulseless VT who did not achieve return of spontaneous circulation (ROSC) after at least three defibrillation attempts were randomly assigned to receive either amiodarone or placebo, with the amiodarone group demonstrating superior outcomes [11]. Conversely, another RCT comparing amiodarone, lignocaine, and placebo among patients with VF or pulseless VT unresponsive to one or more shocks found no significant improvement with amiodarone treatment [12]. Because these RCTs yielded conflicting results, the overall efficacy of amiodarone in this context remains inconclusive. Analyses based on real-world data may therefore help clarify its clinical utility within emergency medical services (EMS) [13]. To date, however, no observational research has specifically assessed the effect of amiodarone administered during prehospital resuscitation using population-based data.

If early administration of amiodarone in the pre-hospital phase were shown to correlate with improved outcomes, its broader implementation in OHCA with shockable rhythms might enhance survival and neurological recovery. Accordingly, this study aimed to evaluate the effectiveness of prehospital amiodarone use in adults with OHCA presenting with shockable rhythms, while appropriately adjusting for resuscitation time bias and time-dependent confounding through real-world registry data.

## Materials and methods

### Study design

We performed a post hoc analysis based on a multi-center, prospective observational registry—the Survey of Survivors after Cardiac Arrest in the Kanto Area 2017 (SOS-KANTO 2017). This registry encompassed 42 hospitals across Japan’s Kanto region and included patients enrolled between September 2019 and March 2021. Both prehospital and in-hospital data were prospectively collected. Prehospital information was recorded by EMS providers, while in-hospital variables were entered by attending physicians at each site through a secure, web-based platform. The registry followed an open-label design, and outcome assessors were not blinded to patient information.

Ethical approval for the SOS-KANTO 2017 registry and the present secondary analysis was obtained from the institutional review boards of all participating centers. This specific analysis was also approved by the Ethics Committee of Jichi Medical University Saitama Medical Center (approval ID: S19-012). Informed consent was waived because all procedures adhered to standard cardiopulmonary resuscitation (CPR) protocols; however, participants were provided with the opportunity to opt out via the website of the Department of Emergency Medicine at Jichi Medical University Saitama Medical Center.

This investigation was conducted in accordance with the ethical principles outlined in the Declaration of Helsinki and its subsequent revisions. Reporting followed the STrengthening the Reporting of Observational Studies in Epidemiology (STROBE) guidelines (Supplementary Table S1) [14].

### Participants

The inclusion criteria were: (1) patients who received resuscitation provided by EMS personnel, (2) transport to the hospital by ground or air ambulance accompanied by a physician, and (3) an initial monitored cardiac rhythm of VF or pulseless VT. In Japan, EMS personnel are not authorized to administer prehospital amiodarone; it can be given only when a physician responds to the scene and accompanies the patient during ground or air transport to the hospital.

The exclusion criteria were as follows: (1) OHCA resulting from trauma, (2) age younger than 18 years, (3) missing or inconsistent data—such as negative time values—for the interval between physician contact and amiodarone administration, and (4) incomplete information regarding covariates used in the Cox regression model or missing outcome data. Details of the covariates included in the analysis are described in the Statistical Analysis section.

### Japanese EMS in OHCA cases

In Japan, national resuscitation guidelines are issued by the Japan Resuscitation Council (JRC) based on the recommendations of the International Liaison Committee on Resuscitation, whereas operational EMS protocols are implemented within the Fire and Disaster Management Agency framework through regional medical-control systems [15]. Each EMS unit typically comprises three personnel, including at least one certified emergency medical technician (EMT) trained in providing prehospital emergency care. EMS providers routinely deliver high-quality basic life support with automated external defibrillator use and employ airway adjuncts (e.g., supraglottic airway devices such as laryngeal tubes) and bag-valve-mask ventilation as permitted. Advanced procedures are scope-dependent; endotracheal intubation is permitted only for EMTs who have completed specialized training and certification, and its use is generally restricted to patients with OHCA.

The range of medications that EMTs can administer is highly limited. Permitted drugs include intramuscular adrenaline for anaphylaxis, intravenous adrenaline for OHCA, intravenous glucose for hypoglycemia, and intravenous crystalloid fluids for shock management, in accordance with local medical-control protocols. Importantly, prehospital administration of amiodarone by EMTs for OHCA is prohibited under current regulations. In addition, EMS personnel are generally not permitted to terminate resuscitation in the field except in cases with obvious signs of death, which differs from practice in the United States and in many European countries.

### Data collection

The following data were collected: age, sex, clinical frailty scale (very fit, well, managing well, vulnerable, mildly frail, moderately frail, severely frail, very severely frail, terminally ill), charlson comorbidity index, time of emergency call, witness status (none, EMS personnel, others), bystander CPR (presence, absence, presence including rescue breathing), initial monitored cardiac rhythm (VF, pulseless VT), cause of cardiac arrest (cardiogenic, respiratory, other intrinsic factors), time from scene to EMS contact, adrenaline administration before physician contact, AAM before physician contact, shock delivery before physician contact, adrenaline administration after physician contact but before amiodarone administration, time from physician contact to adrenaline administration before amiodarone administration, time from scene to amiodarone administration, time from physician contact to amiodarone administration, AAM after physician contact but before amiodarone administration, time from physician contact to AAM before amiodarone administration, prehospital AAM (supraglottic airway device, endotracheal tube), time from EMS contact to hospital arrival, time from scene to ROSC, 30-day cerebral performance category (CPC), and survival [16]. Based on a previous study, the emergency call time was categorised as three intervals (7:00–14:59, 15:00–22:59, 23:00–6:59 h) and AAM includes both supraglottic airway and tracheal intubation, which are considered to have comparable efficacy [17,18].

### Outcome measures

The primary outcome was a favourable 30-day neurological outcome following OHCA, which was defined as a CPC score of 1 or 2 [16]. The secondary outcome was 30-day survival.

### Statistical analyses

Continuous variables were expressed as medians with interquartile ranges (IQRs), whereas categorical variables were summarised as counts and percentages. Missing data were not imputed; thus, analyses were performed using complete cases only. The exposure was amiodarone administration at a given time after physician contact. The comparator was no amiodarone at the same time point (ie, patients who had not yet received amiodarone). Thus, the at-risk group may include patients who later receive amiodarone. Therefore, the comparison reflects amiodarone administration at that time versus no amiodarone, not ‘amiodarone versus never-amiodarone‘.

Initially, a time-dependent propensity score (PS) was derived using a Cox proportional hazards model incorporating both time-varying and fixed covariates, without accounting for competing risks. In this model, amiodarone administration served as the dependent variable, and the following covariates were included as predictors [19,20].

Time-dependent covariates comprised: (1) adrenaline administration after physician contact but prior to amiodarone use, (2) the elapsed time from physician contact to adrenaline administration before amiodarone use, (3) AAM initiated after physician contact but before amiodarone use, and (4) the corresponding interval between physician contact and AAM initiation. Time-invariant covariates included age, sex, time of emergency call, witness status, bystander CPR, etiology of cardiac arrest, time from scene arrival to EMS contact, pre-physician adrenaline administration, pre-physician AAM, pre-physician defibrillation, and transport interval from EMS contact to hospital arrival. Covariates demonstrating no variation (i.e., zero variance) or leading to estimation failure due to complete separation were excluded from the model.

For risk-set matching using time-dependent PS, each patient who received amiodarone was matched with up to three patients who were at comparable risk of receiving it, using a caliper width equal to 0.2 of the standard deviation of the estimated PS [21,22]. Prior evidence indicates that 1:3 matching yields improved precision with minimal increase in bias; therefore, this matching ratio was adopted [23]. Covariate balance was considered adequate when the standardized difference (SD) between groups was below 0.25 [24,25].

Subsequently, a generalized estimating equation (GEE) model with an exchangeable correlation structure was fitted to account for clustering within hospitals. The dependent outcomes were 30-day survival and favorable neurological outcome at 30 days. Effect sizes were presented as risk ratios (RRs) with corresponding 95% confidence intervals (CIs). All statistical analyses were conducted using R software (version 4.1.3; The R Foundation for Statistical Computing, Vienna, Austria). A two-sided p-value < 0.05 was considered statistically significant.

### Sensitivity analysis

To account for possible residual confounding, a sensitivity analysis was conducted using the GEE model. This analysis incorporated explanatory variables originally intended as covariates but showing SDs exceeding 0.25 after time-dependent PSM. In addition, to evaluate the stability of the results obtained with 1:3 time-dependent PSM, we performed a supplementary analysis using a 1:1 matching approach.

## Results

### Patient enrolment

**Fig. 1:**
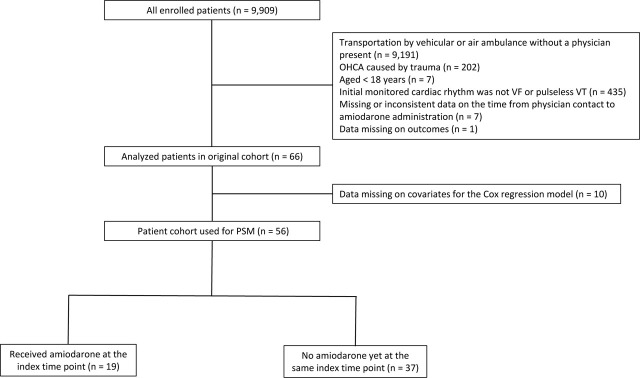
**Flowchart of the screening and enrolment process within the current study.** Exposure was amiodarone administration at each index time point; controls were patients who had not yet received amiodarone at that same index time point (and could receive it later).

Among 9,909 included patients, 56 patients (0.6%) were analyzed as PSM and 19 patients (0.2%) were administered amiodarone at the index time point (Figure 1).

### Patient characteristics

Patient demographics and the extent of missing data in the original cohort are summarized in Table 1. The overall median age (IQR) was 65 (52–76) years, and 54 patients (81.8%) were male. Cardiac arrest was witnessed in 57 cases (86.4%), while 43 patients (65.2%) received bystander-initiated cardiopulmonary resuscitation (CPR). The initial monitored rhythm was ventricular fibrillation in 63 patients (95.5%), and 60 (90.9%) received defibrillation by emergency medical services (EMS) before physician contact. The median time intervals were 27 (22–32) minutes from scene arrival to amiodarone administration, 4 (2–7) minutes from physician contact to amiodarone administration, and 13 (9–16) minutes from physician contact to hospital arrival (Table 1 and 2).

**Table 1.** Characteristics of adults with OHCA with and without amiodarone in original cohort

	**Overall (n = 66)**	**No amiodarone (n = 45)**	**Amiodarone (n = 21)**	**SD**
Age, median (IQR)	65 (52–76)	69 (52–77)	63 (53–71)	0.19
Male, n (%)	54 (81.8)	40 (88.9)	14 (66.7)	0.55

Clinical frailty scale^a^, n (%)				−0.06
Very fit	3 (5.0)	2 (5.0)	1 (5.0)	
Well	9 (15.0)	7 (17.5)	2 (10.0)	
Managing well	29 (48.3)	19 (47.5)	10 (50.0)	
Vulnerable	10 (16.7)	6 (15.0)	4 (20.0)	
Mildly frail	4 (6.7)	2 (5.0)	2 (10.0)	
Moderately frail	2 (3.3)	2 (5.0)	0 (0)	
Severely frail	3 (5.0)	2 (5.0)	1 (5.0)	
Very severely frail	0 (0)	0 (0)	0 (0)	
Terminally ill	0 (0)	0 (0)	0 (0)	
Charlson comorbidity index^b^, median (IQR)	0 (0–1)	0 (0–1)	0 (0–1)	0.38

Time of emergency call, n (%)				0.42
7:00–14:59	32 (48.5)	21 (46.7)	11 (52.4)	
15:00–22:59	25 (37.9)	16 (35.6)	9 (42.9)	
23:00–6:59	9 (13.6)	8 (17.8)	1 (4.8)	

Witness status, n (%)				0.25
None	8 (12.1)	6 (13.3)	2 (9.5)	
EMS personnel	1 (1.5)	1 (2.2)	0 (0)	
Others	57 (86.4)	38 (84.4)	19 (90.5)	

Bystander CPR, n (%)				0.47
Presence	43 (65.2)	29 (64.4)	14 (66.7)	
Absence	23 (34.8)	16 (35.6)	7 (33.3)	

Initial monitored cardiac rhythm, n (%)				0.38
VF	63 (95.5)	42 (93.3)	21 (100)	
Pulseless VT	3 (4.5)	3 (6.7)	0 (0)	

Cause of cardiac arrest, n (%)				0.16
Cardiogenic	61 (92.4)	41 (91.1)	20 (95.2)	
Other intrinsic disease	5 (7.6)	4 (8.9)	1 (4.8)	
Time from scene to initiation of CPR^c^, min, median (IQR)	11 (9–13)	11 (10–14)	10 (9–12)	0.17
Adrenaline administration by EMS before physician contact^d^, n (%)	34 (68.0)	21 (70.0)	13 (65.0)	0.31
AAM by EMS before physician contact, n (%)	39 (59.0)	25 (55.5)	14 (66.6)	0.39

Type of AAM by EMS, n (%)				0.39
Supraglottic airway device	36 (54.5)	24 (53.3)	12 (57.1)	
Endotracheal tube	3 (4.5)	1 (2.2)	2 (9.5)	
Shock delivery by EMS before physician contact, n (%)	60 (90.9)	41 (91.1)	19 (90.5)	0.16
Adrenaline administration before amiodarone administration, n (%)	24 (36.4)	16 (35.6)	8 (38.1)	0.05
Time from physician contact to adrenaline administration before amiodarone administration^e^, min, median (IQR)	3 (1.8–4.3)	3.5 (1.8–5.3)	2.5 (1.8–3)	0.39
AAM before amiodarone administration, n (%)	24 (36.4)	16 (35.6)	8 (38.1)	0.05
Time from physician contact to AAM before amiodarone administration^f^, min, median (IQR)	3 (3–5)	4.5 (3–5)	3 (2.8–3)	0.84
Time from scene to amiodarone administration, min, median (IQR)	27 (22–32)	-	27 (22–32)	-
Time from physician contact to amiodarone administration, min, median (IQR)	4 (2–7)	-	4 (2–7)	-
Time from physician contact to hospital arrival, min, median (IQR)	13 (9–16)	14 (8–16)	11 (10–15)	−0.18
Time from scene to ROSC^g^, min, median (IQR)	27 (21–38)	26 (21–37)	28 (23–49)	−0.34

Missing data:

a n = 6 (9.1%);

b n = 2 (3.0%);

c n = 8 (12.1%);

d n = 17 (25.8%);

e n = 42 (63.6%);

f n = 42 (63.6%);

g n = 23 (34.8%)

Abbreviations: AAM, advanced airway management; CPR, cardiopulmonary resuscitation; IQR, interquartile range; EMS, emergency medicine service; OHCA, out-of-hospital cardiac arrest; ROSC, return of spontaneous circulation; SD, standardized difference; VF, ventricular fibrillation; VT, ventricular tachycardia.

**Table 2.** Outcomes in original cohort

	**Overall (n = 66)**	**No amiodarone (n = 45)**	**Amiodarone (n = 21)**	**SD**
30-day neurological outcome				
CPC 1	10 (15.2)	9 (20.0)	1 (4.8)	0.67
CPC 2	5 (7.6)	3 (6.7)	2 (9.5)	
CPC 3	3 (4.5)	1 (2.2)	2 (9.5)	
CPC 4	7 (10.6)	6 (13.3)	1 (4.8)	
CPC 5	41 (62.1)	26 (57.8)	15 (71.4)	
30-day favorable neurological outcome	15 (22.7)	12 (26.7)	3 (14.3)	0.31
30-day survival	25 (37.9)	19 (42.2)	6 (28.6)	0.29

Abbreviations: CPC, cerebral performance category; SD, standardized difference.

Several covariates were excluded from the Cox regression model due to lack of variability (i.e., zero variance) or complete separation that prevented coefficient estimation. These variables included witness status, bystander CPR, cause of cardiac arrest, adrenaline administration before physician contact, and AAM prior to physician contact. Following time-dependent PSM, the following variables could not be balanced to achieve a standardized difference < 0.25: adrenaline administration by EMS before physician contact, defibrillation by EMS before physician contact, adrenaline administration prior to amiodarone administration, AAM prior to amiodarone administration, and time from EMS contact to hospital arrival (Table 2). Among the cohort, 19 patients (0.2%) were administered amiodarone at the index time point, 21 patients (27.6%) received adrenaline before amiodarone, and the same proportion underwent AAM prior to amiodarone use. The median time from physician contact to adrenaline administration (before amiodarone) was 3 (2–3) minutes, and the interval from physician contact to AAM (before amiodarone) was likewise 3 (2–3) minutes. The median time from physician contact to hospital arrival was 14 (11–17) minutes (Table 3).

**Table 3.** Characteristics of adults with OHCA with and without amiodarone after time-dependent propensity score matching

	**Overall (n = 76)**	**No amiodarone yet at the same index time point (n = 57)**	**Received amiodarone at the index time point (n = 19)**	**SD**
Age, median (IQR)	63 (52–76)	59 (52–80)	65 (57–73)	−0.01
Male, n (%)	53 (69.7)	41 (71.9)	12 (63.2)	0.19

Clinical frailty scale^a^, n (%)				−0.13
Very fit	2 (2.8)	1 (1.9)	1 (5.6)	
Well	16 (22.5)	15 (28.3)	1 (5.6)	
Managing well	30 (42.3)	21 (39.6)	9 (50.0)	
Vulnerable	14 (19.7)	10 (18.9)	4 (22.2)	
Mildly frail	2 (2.8)	0 (0)	2 (11.1)	
Moderately frail	7 (9.9)	6 (11.3)	1 (5.6)	
Severely frail	2 (2.8)	1 (1.9)	1 (5.6)	
Very severely frail	0 (0)	0 (0)	0 (0)	
Terminally ill	0 (0)	0 (0)	0 (0)	
Charlson comorbidity index, median (IQR)	0 (0–1)	0 (0–0)	0 (0–1)	0.05

Time of emergency call, n (%)				0.10
7:00–14:59	39 (51.3)	29 (50.9)	10 (52.6)	
15:00–22:59	34 (44.7)	26 (45.6)	8 (42.1)	
23:00–6:59	3 (3.9)	2 (3.5)	1 (5.3)	

Witness status, n (%)				0
None	0 (0)	0 (0)	0 (0)	
EMS personnel	0 (0)	0 (0)	0 (0)	
Others	76 (100)	57 (100)	19 (100)	

Bystander CPR, n (%)				0.16
Presence	56 (73.7)	43 (75.4)	13 (68.4)	
Absence	20 (26.3)	14 (24.6)	6 (31.6)	
Initial monitored cardiac rhythm, n (%)				0
VF	76 (100)	57 (100)	19 (100)	
Pulseless VT	0 (0)	0 (0)	0 (0)	

Cause of cardiac arrest, n (%)				0.09
Cardiogenic	73 (96.1)	55 (96.5)	18 (94.7)	
Other intrinsic disease	3 (3.9)	2 (3.5)	1 (5.3)	
Time from scene to initiation of CPR, min, median (IQR)	11 (10–12)	11 (10–13)	10 (9–12)	0.10
Adrenaline administration by EMS before physician contact^b^, n (%)	44 (74.6)	32 (78.0)	12 (66.7)	0.26
AAM by EMS before physician contact, n (%)	49 (64.5)	36 (63.1)	13 (68.5)	0.22

Type of AAM by EMS, n (%)				0.22
Supraglottic airway device	44 (57.9)	32 (56.1)	12 (63.2)	
Endotracheal tube	5 (6.6)	4 (7.0)	1 (5.3)	
Shock delivery by EMS before physician contact, n (%)	71 (93.4)	54 (94.7)	17 (89.5)	0.33
Adrenaline administration before amiodarone administration, n (%)	21 (27.6)	14 (24.6)	7 (36.8)	0.27
Time from physician contact to adrenaline administration before amiodarone administration^c^, min, median (IQR)^*^	3 (2–3)	3 (2.3–3)	2 (1.5–3)	0.67
AAM before amiodarone administration, n (%)	21 (27.6)	14 (24.6)	7 (36.8)	0.27
Time from physician contact to AAM before amiodarone administration^d^, min, median (IQR)^*^	3 (2–3)	3 (2.3–3.8)	3 (2.5–3)	0.24
Time from physician contact to hospital arrival, min, median (IQR)	14 (11–17)	14 (11–17)	11 (10–15)	0.28
Time from scene to ROSC^e^, min, median (IQR)	25 (17–35)	25 (17–28)	28 (23–49)	−0.51

Missing data:

a n = 5 (6.6%);

b n = 17 (22.4%);

c n = 55 (72.4%);

d n = 55 (72.4%);

e n = 18 (23.7%).

Abbreviations: AAM, advanced airway management; CPR, cardiopulmonary resuscitation; IQR, interquartile range; EMS, emergency medicine service; OHCA, out-of-hospital cardiac arrest; ROSC, return of spontaneous circulation; SD, standardized difference; VF, ventricular fibrillation; VT, ventricular tachycardia.

* Matching does not necessarily improve the balance, since the variable is only for patients who received that treatment.

### Analyses involving timing variables and GEE

Amiodarone administration was not associated with favourable 30-day neurological outcomes (RR [95% CI]: 0.45 [0.14–1.47]) and 30-day survival (RR [95% CI]: 0.74 [0.31–1.73]), respectively (Table 4).

**Table 4.** Outcomes in time-dependent propensity score matched cohort

**Outcomes**	**Number of patients with outcome/total number of patients (%)**		**Risk ratio (95% CI)**

	**No amiodarone yet at the same index time point**	**Received amiodarone at the index time point**	
30-day neurological outcome			
CPC 1	12 (21.1)	1 (5.3)	
CPC 2	6 (10.5)	2 (10.5)	
CPC 3	1 (1.8)	2 (10.5)	
CPC 4	1 (3.5)	1 (5.3)	
CPC 5	36 (63.2)	13 (68.4)	
30-day favorable neurological outcome	18/57 (31.6)	3/19 (15.8)	0.45 (0.14–1.47)
30-day survival	21/57 (36.8)	6/19 (31.6)	0.74 (0.31–1.73)

Abbreviations: CI, confidence interval.

### Sensitivity analysis

A sensitivity analysis was conducted using the GEE model, which included the following covariates: adrenaline administration by EMS before physician contact, defibrillation by EMS prior to physician contact, adrenaline administration before amiodarone use, AAM before amiodarone administration, and the time interval from EMS contact to hospital arrival. In this model, prehospital amiodarone use showed no significant association with a favorable 30-day neurological outcome (RR, 0.92; 95% CI, 0.29–2.89). However, model convergence could not be achieved, and results for 30-day survival were therefore unavailable (Table 4).

In the additional analysis using 1:1 time-dependent propensity score matching, amiodarone administration likewise demonstrated no association with either favorable 30-day neurological outcome (RR, 1.00; 95% CI, 0.26–3.83) or 30-day survival (RR, 1.60; 95% CI, 0.53–4.86) (Table 4).

**Table 5.** Results of sensitivity analysis

**Outcomes (RR [95%CI])**	**GEE results including covariates with SD > 0.25 in time-dependent PSM**	**1:1 time-dependent PSM**
30-day favorable neurological outcome	0.92 (0.29–2.89)	1 (0.26–3.83)
30-day survival	-^*^	1.6 (0.53–4.86)

Abbreviations: CI, confidence interval; GEE, generalised estimating equation; PSM, propensity score matching; RR, risk ratio; SD, standardised difference.

* The model did not converge when performing the GEE analysis, and results for 30-day survival could not be obtained.

## Discussion

In analyses that adjusted for both resuscitation time bias and time-dependent confounding using real-world data in Japan, early prehospital administration of amiodarone, compared with no amiodarone at the corresponding time point during prehospital resuscitation, was not associated with improved 30-day neurological outcomes or survival.

The two prior RCTs assessing the efficacy of prehospital amiodarone for shock-refractory out-of-hospital cardiac arrest (OHCA), when considered alongside the present findings, offer several possible interpretations [11,12]. Around the year 2000, the American Heart Association (AHA) guidelines for CPR followed the “Airway–Breathing–Circulation (ABC)” sequence, recommending amiodarone after three failed defibrillation attempts in patients with VF or pulseless VT. [26] However, the sequence was revised to “Circulation–Airway–Breathing (CAB)” in 2005, and by 2010, the recommendation for three consecutive shocks in shock-refractory VF/pulseless VT was removed [27,28]. The positive findings of the 1999 RCT may therefore reflect differences in historical resuscitation protocols and cannot be directly generalized to current clinical settings [11].

By contrast, global resuscitation practices during the period of the 2016 RCT were broadly similar to those in Japan when the present data (2019–2021) were collected [12]. Both the 2016 trial and the current study failed to demonstrate a survival benefit from prehospital amiodarone administration [12]. The 2016 RCT included patients with a mean age of 64 years, of whom 71% had witnessed arrests and 61% received bystander CPR, with an average interval of 8 minutes from emergency call to arrival of advanced life support personnel [12]. In our cohort, the median age was 63 years, all arrests were witnessed, 74% received bystander CPR, and the median time from scene arrival to initiation of CPR was 11 minutes. Given these parallels in patient profiles, the similar findings between studies likely reflect comparable populations [12]. Furthermore, although our study has inherent limitations, the reproducibility of the results across independent datasets supports a certain degree of robustness [12].

From a physiological standpoint, the lack of benefit from prehospital amiodarone can also be interpreted in relation to the temporal evolution of VF/pulseless VT. These rhythms are thought to progress through three phases following collapse: the electrical phase (within approximately 4 minutes), the circulatory phase (4–10 minutes), and the metabolic phase (beyond 10 minutes) [29]. Once the metabolic phase begins, myocardial responsiveness to pharmacologic agents declines significantly.[29] In the 2016 RCT, the mean interval from scene arrival to amiodarone administration was 19 minutes, while in our study it was 27 minutes [12]. In both cases, administration occurred well beyond the 10-minute threshold, suggesting that most patients were likely in the metabolic phase, thereby reducing the potential effectiveness of amiodarone [12,29].

However, it is important to reiterate that our findings were obtained in Japan, where the scope of prehospital EMS interventions and the use of field termination of resuscitation (TOR) are highly restricted. Indeed, in our study, applying an inclusion criterion requiring transport to the hospital by ground or air ambulance accompanied by a physician led to the exclusion of 9,191 patients, and only 19 patients (0.2%) received prehospital amiodarone. In the United States, prehospital EMS care and field TOR are substantially more permissive, and the AHA guidelines provide a weak recommendation to “consider” amiodarone for shock-refractory VF or pulseless VT [30]. In contrast, Europe comprises a mix of countries with both restrictive and more permissive prehospital EMS systems and field TOR practices. The European Resuscitation Council guidelines describe the use of amiodarone for shock-refractory VF/pulseless VT that persists after three shocks, but do not explicitly state a recommendation strength [31]. In Japan, the JRC guidelines weakly recommend amiodarone for shock-refractory VF/pulseless VT; however, its use is feasible only in very limited circumstances—specifically, when a physician responds to the scene via a doctor car or physician-staffed helicopter [32]. Thus, substantial cross-national differences in prehospital systems must be considered when interpreting our results. Accordingly, because our inclusion criterion required transport to hospital by ground or air ambulance accompanied by a physician, the eligible sample was very small; as a result, limited statistical power may have precluded a valid assessment of the efficacy of prehospital amiodarone in the current study. Details of the sample size and power calculations are provided in the limitations section below.

This investigation is, to our knowledge, the first to evaluate the prognostic impact of prehospital amiodarone for shockable OHCA while accounting for resuscitation time bias and time-dependent confounding using real-world registry data. Early prehospital administration of amiodarone was not associated with improved outcomes compared with no amiodarone at the corresponding time point during prehospital resuscitation in the current retrospective observational cohort. Given the potential for residual confounding, selection mechanisms, and health-system constraints that influence both treatment delivery and timing, these findings should not be interpreted as evidence to withhold guideline-directed antiarrhythmic therapy. Rather, they highlight the need for further research to clarify whether very early administration—ideally within 10 minutes of cardiac arrest recognition—confers benefit, using large, well-designed prospective comparative-effectiveness studies and, where feasible, pragmatic interventional trials, with RCTs representing the ideal design to establish causality when practical and ethically feasible.

Several limitations must be considered. First, the generalizability of our findings may be constrained because the dataset was limited to institutions in Japan’s Kanto region, an urban area characterized by advanced emergency care infrastructure [33]. The median interval from physician contact to hospital arrival was 13 minutes, which is considerably shorter than the approximately 66 minutes reported in rural settings [34]. Thus, the results may differ in less urbanized areas. In addition to concerns about geographic generalizability, our study applied an inclusion criterion requiring transport to the hospital by ground or air ambulance accompanied by a physician, which resulted in the exclusion of 9,191 patients. Because prehospital EMS interventions are highly restricted in Japan, this inclusion criterion led to a large number of exclusions. Given that the overall cohort comprised 9,909 patients, this represents a very large proportion of exclusions, and selection bias may have reduced the external validity of our findings. Moreover, because prehospital EMS interventions are limited in Japan, only 19 patients (0.2%) received pre-hospital amiodarone, which may have contributed not only to reduced external validity but also to limited statistical power, as discussed in the third limitation. Second, because Japanese emergency medical technicians (EMTs) are legally prohibited from administering amiodarone in the prehospital setting, extrapolation of these findings to countries with broader prehospital pharmacologic protocols may be limited. Third, the lack of a statistically significant benefit may reflect insufficient sample size rather than true ineffectiveness, as only 21 patients in our dataset received prehospital amiodarone. With a larger cohort, a clinically meaningful difference might emerge. Using the proportions of favorable neurological outcome in the two groups in the original cohort, we calculated the sample size that would be required for a 1:1 RCT with 80% power and a two-sided α of 0.05. The estimated sample size was 182 patients per group (364 in total). Therefore, the current study was clearly underpowered due to an insufficient sample size. Additionally, in the sensitivity analysis that incorporated variables with standardized differences > 0.25 after propensity score matching, the small number of cases may have increased the risk of model overfitting. Lastly, in SOS-KANTO 2017, the number of amiodarone administrations was recorded, but the number of observations was too small to incorporate this information into our analyses. In addition, because the dose per administration was not recorded, we were unable to perform dose-specific analyses, although amiodarone was likely administered as the standard 300-mg bolus per dose according to Japanese resuscitation protocols. Given that higher doses of antiarrhythmic agents are typically associated with greater efficacy, the absence of adjustment for frequency or dosage may have influenced the precision of our estimates.

## Conclusions

The current study did not demonstrate the efficacy of prehospital amiodarone in OHCA with shockable rhythms, even after adjustment for resuscitation time bias and time-dependent confounding using real-world data from urban areas in Japan. These findings should be interpreted as descriptive associations under specific system constraints and should not be taken as evidence to withhold guideline-directed therapy. However, the number of eligible patients in our study was very small, and further prospective studies are warranted, particularly to evaluate the impact of much earlier drug administration.

## Supplementary Material

Supplementary Material Details I

Supplementary Material Details II

## Abbreviations

OHCA: out-of-hospital cardiac arrest;
PSM: propensity score matching;
VF: ventricular fibrillation;
VT: pulseless ventricular tachycardia.
