# Supplementary material for: Efficacy of prehospital amiodarone on survival in adult out-of-hospital cardiac arrest: a retrospective observational study
Source: J Crit Care Med (Targu Mures). 2026 Jul 27;12(3):426–37. doi: 10.62838/jccm-2026-0024 (PMC13403018; doi:10.62838/jccm-2026-0024)
Supplement: Supplementary file 2 — Supplementary Material Details II [file jccm-2026-0024_Supplementary2.pdf]

Contributors

SOS-KANTO 2017 Steering Council

Tokyo Women's Medical University (Munekazu Takeda), Kimitsu Chuo Hospital (Nobuya Kitamura), Chiba University Hospital (Taka-aki Nakada), The University of Tokyo (Hideo Yasunaga , Shotaro Aso), Nippon Medical School Musashikosugi Hospital (Takashi Tagami), Chiba Kaihin Municipal Hospital (Yosuke Honma, Yosihisa Tateishi), Nippon Medical School Hospital (Tomoko Ogasawara), Keio University Hospital (Kei Hayashida), Tokyo Bay Urayasu/Ichikawa Medical Center (Hiraku Funakoshi), Juntendo University Nerima Hospital (Tomohisa Nomura), Tokyo Dental College Ichikawa General Hospital (Masaru Suzuki), Tokyo Metropolitan Bokutoh Hospital (Kazuhiro Sugiyama), Nihon University Hosapital (Atsushi Sakurai)

SOS-KANTO 2017 Study Group

Tokai University School of Medicine (Yoshihide Nakagawa); St.Marianna University School of Medicine, Yokohama Seibu Hospital (Minoru Yoshida); Saitama Medical Center Department of Emergency Medicine(ER) (Masaki Hisamura); Kawasaki Municipal Hospital Emergency and Critical Care Center (Kunio Kanao); Japanese Red Cross Maebashi Hospital (Jun Maruyama); Juntendo University Urayasu Hospital (Tadashi Ishihara); Tokyo Women’s Medical University Hospital (Munekazu Takeda); Kimitsu Chuo Hospital (Nobuya Kitamura); Chiba University Graduate School of Medicine (Taku Oshima); Dokkyo Medical University Saitama Medical Center (Daisuke Sugimoto); National Disaster Medical Center (Mayuko Kaneko); Nihon University Hospital (Atsushi Sakurai); Nippon Medical School Tamanagayama Hospital (Chie Tanaka); Tokyo Women’s Medical University Yachiyo Medical Center (Tomohito Sadahiro); Japanese Red Cross Medical Center (Yuta Moroe); National Hospital Organization Mito Medical Center (Yusuke Tsutsumi); Tokyo Metropolitan Tama Medical Center (Tomohide Koyama); Gunma University Graduate School of Medicine (Kazunori Fukushima MD); Saitama Red Cross Hospital (Kazuya Kiyota); Tokyo Metropolitan Bokutoh Hospital (Kazuhiro Sugiyama); Keio University Hospital (Ryo Yamamoto); Teikyo University School of Medicine (Ryuichi Nishi); National Center for Global Health and Medicine Hospital (Makiko Yamamoto); Medical Hospital of Tokyo Medical and Dental University (Naoshi Urushibata); Juntendo University Nerima Hospital (Hiroki Takami); Nihon University Itabashi Hospital (Nami Sawada); National Center for Child Health and Development (Shima Ohnishi); Chiba Aoba Municipal Hospital (Shunsuke Otani); Matsudo City Hospital (Masayuki Yagi); Japanese Red Cross Narita Hospital (Yoshihisa Tateishi); Tokyo Bay Urayasu/Ichikawa Medical Center (Yosuke Honma); OTA Memorial Hospital (Kazuki Akieda); Tokyo Dental College Ichikawa General Hospital (Masaru Suzuki) ; Tokyo The Jikei University Kashiwa Hospital (Izumu Hasegawa); Jichi Medical University Saitama Medical Center (Masahiro Kashiura MD); IUHW Narita Hospital (Ryuhei Igeta); University of Tsukuba Hospital (Yasuaki

Koyama MD); Seirei Hamamatsu General Hospital (Takahiro Atsumi); Seirei Mikatagahra General Hospital (Go Makishi); Eastern Chiba Medical Center (Tomoaki Hashida); Nagoya University Hospital (Yuma Yasuda) (http://www.jaam-kanto.jp/sos_kanto/sos_kanto2017_contributors.html)
